# United States Marine Hospital

**Published:** 1855-08

**Authors:** Chas. A. Davis, Alph. B. Crosby

**Affiliations:** Superintendent; Chelsea, Mass.; House Surgeon; Chelsea, Mass.


From the New Hampshire Journal of Medicine.

*United States Marine Hospital.* Dr. CHAS. A. DAVIS, Superintendent. ALPH. B. CROSBY, House Surgeon.

One of the most common diseases among seafaring men is the chronic dysentery, acquired on the east coast of Africa. This is technically known among the sailors as the "coast dysentery," and is justly regarded as the bane of that locality. Few of those who visit those shores escape without suffering more or less from this distressing malady. The north-western coast of Africa is infested with various miasmatic diseases, among which, perhaps, the most prominent is bilious remittent fever. The diseases of this portion of the country are not always, nor even generally, complicated with diarrhœa, but on the south-western coast dysentery is one, if not *the* disease to be feared. Much of this is due to the climate, and much, undoubtedly, is to be attributed to the influence of malaria. The thermometer during the twenty-four hours frequently ranges from 60 to 100°, and even higher. The most intense heat usually commences between ten and eleven o'clock, A.M., and continues, with only slight abatement, until sun-down. Immediately after sunset the temperature is reduced with great rapidity until the thermometer stands in the vicinity of 60°. This sudden transition in the temperature of 40° is itself a strong predisposing cause of disease. The fall of dew is very copious—so much so as to saturate the clothes of those who are exposed to it. The heat is so intense that the pitch frequently melts and runs from the beams, and the decks must be constantly wet to prevent their cracking. The subsequent reduction of temperature coming as it does so suddenly, cannot fail to prove deleterious.

The class of men who visit this coast are much given to excess in drink and venery. With such predisposing and exciting courses, few persons escape the disease. The disease is sometimes ushered in with a chill—sometimes not; the pulse is accelerated, the tongue slightly coated, and general febrile symptoms ensue. There is always tenesmus, and the stools are bloody and slimy. The frequency of the stools is usually very great, as many even as fifty in the twenty-four hours. Great emaciation ensues, and the face acquires a sallow and cadaverous hue. The liver almost always becomes involved in the course of the disease, and hepatic abscess is of no unfrequent occurrence. An enormous appetite is an accompaniment of the acute stage of the disorder, which, if gratified, creates a sensation of distress in the epigastric region, and aggravates the complaint. After a longer or shorter time, in the acute form, there is usually a lull in all the symptoms; the discharges diminish, the strength improves, and nature seems to make an attempt at recuperation. The improvement is only temporary, the violence of the symptoms soon returns, the emaciation becomes extreme, the feet and legs are frequently anasarcous, and death results from asthenia. This, then, in brief, is the course and termination of the disease if left to itself. It may occupy a few days, a few months, or a few years. Most of the patients are admitted into this hospital after the disease has become chronic, and the time occupied in recovering will range on an average from two months to two years and upward, providing they are subjected to the proper mode of treatment. The lesions in this disorder are quite constant; traces and effects of inflammation exist in the colon, and in many cases in the rectum; also patches of ulceration are found throughout the large intestine and caecum; the mucous membrane is injected in various places; pseudo membranous matter is found in shreds and layers. The mucous membrane is sometimes disorganized, even to the extent of complete sphacelus, perforation frequently exists, having been the immediate cause of death. Evidences of recent gastritis sometimes are apparent. The treatment is very simple, much depending on a well regulated diet. Boiled milk and bread is the diet furnished for these patients in this institution, and the amelioration following this course is very marked. Laxatives employed occasionally, with opium and ipecacuanha, are frequently of great service. Among the alteratives, sulphate of copper, balsam copaiba and oil of turpentine, et cetera, are in many cases efficacious. The Germans lay great stress on injections of nitrate of silver, a flexible tube being introduced as far as it can be conveniently, and a strong solution thrown up. The great majority of patients with this disease recover. The treatment which has seemed most successful here has been a well regulated diet rigidly enforced, and pills containing a grain of opium with a third of a grain of sulphate of copper. This, with a careful attention to the condition of the skin, liver, &c., proves successful in a great majority. I am induced to cite a single case, which, although a fatal one, possesses some points of interest, and shows the result of improper treatment in chronic dysentery. This patient was discharged from the hospital three weeks previously, apparently cured, was advised to pursue a tonic course, and avoid all error in diet This advice was not followed, and subsequently applying to a physician, he was advised to drink freely of alcoholic stimulants. I am informed by the person who furnished him, that the quantity of brandy, wines, &c., which he daily consumed, were enormous. On his admission, he complained of violent gastritis, and had deep seated pains over the kidneys, and liver. The discharges from the bowels were not profuse. During the last few days of his life, he was unable to retain anything in his stomach. In addition to his disease, he had to contend with that terrible depressive despair. Even the non-medical Byron was aware of its malignity, and has well expressed it,

> " Despair of all recovery sp[illegible]ils longevity,
> And makes men's mis'ries of alarming brevity."

The invariable remark of this patient was, " I shall go out feet first." He did go out that way, on the morning of May 28. An autopsy 36 hours after death, presented the following appearances : the whole mucous surface of the stomach in various stages of inflammation, a large part of a slate color; in the base of the stomach a perforation the size of a half dollar, the edges of which were entirely healed, with the exception of a distance of three or four lines. This portion of the stomach was healed to the intestine just below; traces of old inflammation in the colon, mucous coat thickened, but presenting generally a healthy appearance; kidneys hypertrophied and showing fully degeneration; liver weighed nearly six pounds; postero superior portion, adherent to the costal wall; liver being removed a portion remained adhered, the inter-substances soft and easily broken. Mr. Haskall the apothecary at this hospital, (to whom I am indebted for many interesting facts with regard to the " coast" and its diseases) was himself afflicted with this disease for a period ol twenty-eight months. He has now entirely recovered. There is at present in one of the wards, a sailor who, on his admission a month since, had eight and forty discharges in the four and twenty hours, anasarca of the feet and legs, and almost frightful debility. His discharges now number only six in the four and twenty hours, strength much improved, and his limbs no longer anasarcous. This patient is half idiotic, had he known enough he would probably have died. With him however,

> " Ignorance is bliss."

and he is now rapidly recovering.

Chelsea, Mass., June, 1855.

